# TNF signalling drives expansion of bone marrow CD4^+^ T cells responsible for HSC exhaustion in experimental visceral leishmaniasis

**DOI:** 10.1371/journal.ppat.1006465

**Published:** 2017-07-03

**Authors:** Ana Isabel Pinto, Najmeeyah Brown, Olivier Preham, Johannes S. P. Doehl, Helen Ashwin, Paul M. Kaye

**Affiliations:** Centre for Immunology and Infection, Dept. of Biology and Hull York Medical School, University of York, Heslington, York, United Kingdom; University of São Paulo FMRP/USP, BRAZIL

## Abstract

Visceral leishmaniasis is associated with significant changes in hematological function but the mechanisms underlying these changes are largely unknown. In contrast to naïve mice, where most long-term hematopoietic stem cells (LT-HSCs; LSK CD150^+^ CD34^-^ CD48^-^ cells) in bone marrow (BM) are quiescent, we found that during *Leishmania donovani* infection most LT-HSCs had entered cell cycle. Loss of quiescence correlated with a reduced self-renewal capacity and functional exhaustion, as measured by serial transfer. Quiescent LT-HSCs were maintained in infected RAG2 KO mice, but lost following adoptive transfer of IFNγ-sufficient but not IFNγ-deficient CD4^+^ T cells. Using mixed BM chimeras, we established that IFNγ and TNF signalling pathways converge at the level of CD4^+^ T cells. Critically, intrinsic TNF signalling is required for the expansion and/or differentiation of pathogenic IFNγ^+^CD4^+^ T cells that promote the irreversible loss of BM function. These findings provide new insights into the pathogenic potential of CD4^+^ T cells that target hematopoietic function in leishmaniasis and perhaps other infectious diseases where TNF expression and BM dysfunction also occur simultaneously.

## Author summary

Visceral leishmaniasis (VL) is a chronic often fatal disease caused by the protozoan parasites *Leishmania donovani* and *L*. *infantum*. Progressive disease in humans and in animal models is associated with parasite replication at systemic sites, including the bone marrow (BM) and results in significant changes in hematological profile. The mechanisms underlying hematologic dysregulation during infection are largely unknown. Using a panel of stem cell markers, we characterized murine haematopoietic stem and progenitor cells in the BM over the course of *L*. *donovani*-infection in C57BL/6 (B6) mice. Most long-term hematopoietic stem cells (LT-HSCs) in naïve mice are found in a quiescent state, representing cells with the highest degree of reconstitution potential. In contrast, during *L*. *donovani* infection, most LT-HSCs had entered cell-cycle and this correlated with a reduced potential to engraft into syngeneic recipients. HSC exhaustion and other alterations in the hematopoietic compartment did not occur in infected immunodeficient mice, but adoptive transfer of IFNγ-sufficient CD4^+^ T cells restored this phenotype. Using mixed BM chimeras, we established that IFNγ signalling and TNF signalling pathways converge at the level of BM CD4^+^ T cells, with intrinsic TNF signalling being critical for the expansion / differentiation of CD4^+^ T cells that are responsible for HSC exhaustion. Contrary to commonly held views, in the setting of experimental visceral leishmaniasis neither IFNγ nor TNF signalling in HSCs was required for their functional exhaustion. Hence, pro-inflammatory cytokines commonly associated with host protection in leishmaniasis and many other infectious diseases can also drive the development of pathogenic CD4^+^ T cells that cause long term irreversible alterations in HSC function.

## Introduction

T cells reside in bone marrow (BM) and comprise 4–8% of total BM cells. Recent studies have indicated that the BM is a preferential site for homing and persistence of memory T cells that have a high proliferative potential following second encounter with a cognate antigen [1, 2]. Furthermore, alterations in BM T cells have been reported in patients suffering from BM failure syndromes [3] and in experimental models for aplastic anaemia [4, 5]. However, the function(s) of the BM T cell compartment are relatively poorly understood compared to their counterparts in lymphoid tissues, particularly so in the context of infectious diseases where pathogens themselves reside in the BM. An association between alterations in hematopoietic function and changes in BM T cells has been described in mice infected with *Ehrlichia muris* [6, 7], but mechanistic insight into these processes has been limited.

Hematopoiesis is a strictly regulated process that depends on a small pool of Long-term hematopoietic cells (LT-HSCs), which have self-renewal capacity and the potential to give rise to all mature blood cells during the lifespan of an individual. According to the classical pathway of hematopoiesis, LT-HSCs differentiate into short-term hematopoietic stem cells (ST-HSCs) that differentiate into a heterogeneous group of multipotent progenitors (MPPs). LT-HSCs, ST-HSCs and MPPs are contained within the LSK population, so called for their lack of expression of mature blood cell-associated markers (Lineage negative) and their expression of Sca1 and cKit. MPPs give rise to intermediary progenitors, the common lymphoid progenitors (CLPs) and the common myeloid progenitors (CMPs), the latter subsequently giving rise to both granulocyte/macrophage progenitors (GMPs) and megakaryocytic/erythrocyte progenitors (MEPs) [8]. Non-committed and lineage-committed progenitors are collectively defined as hematopoietic stem and progenitor cells (HSPCs).

The integration of systemic and local signals by HSPCs has been suggested to be one mechanism that allows these cells to respond to infection and subsequently help regulate immune effector function [9]. In contrast, prolonged activation and proliferation of HSCs has been associated with functional exhaustion in several infection models [7, 10, 11], and may underlie the association between chronic infection and hematological dysfunction, as commonly described in humans [12]. The immune mechanisms associated with HSC exhaustion and whether these operate in an HSC-intrinsic manner or reflect alterations in the BM microenvironment remain important unanswered questions.

Visceral leishmaniasis (VL), caused by infection with the obligate intracellular parasites *Leishmania donovani* and *L*. *infantum* is characterized by parasite accumulation in systemic tissues, including BM, and clinical signs including hypergammaglobulinaemia, hepato-splenomegaly and disturbances in blood homeostasis, including anemia, thrombocytopenia, leucopenia and neutropenia [13–15]. The infection is fatal without drug treatment and even treated patients may die from bleeding or opportunistic bacterial infections [16, 17]. In humans, splenic sequestration and ineffective haematopoiesis have been suggested as possible causes to explain peripheral cytopenia, and noted alterations in the BM include erythroid hyperplasia, increased plasma cells, increased frequency of granulocytic and megakaryocyte immature forms, and histiocytic hyperplasia [15]. Furthermore, several clinical reports have described pancytopenia in VL patients followed by a multilineage myelodysplasia reminiscent of true myelodysplastic syndrome (MDS), suggesting the presence of ineffective haematopoiesis [18–20].

Experimental rodent models have been extensively used to study the immunopathology of VL, also reporting alterations in hematopoietic function. For example, *L*. *donovani* infection in BALB/c mice is associated with increased numbers of hematopoietic precursor cells, as assessed by colony-forming units in culture [21], and both mice and hamsters show various degrees of cytopenia and changes in BM cellularity following infection [22, 23]. Here, we demonstrate that *L*. *donovani* infection in mice drives LT-HSCs into active proliferation at the expense of cells in quiescence, leading to functional exhaustion. Importantly, this response was dependent upon increased numbers of IFNγ-producing CD4^+^ T cells with resident effector function in the BM of infected mice, but not on HSC-intrinsic IFNγ signalling. Unexpectedly, we found that expansion of BM effector T cells was regulated by T cell-intrinsic TNF receptor signalling, indicating a novel means by which TNF and IFNγ signalling pathways cooperate and converge at the level of CD4^+^ T cells to effect long-lasting impairment of hematopoietic function during infection.

## Results

### *L*. *donovani* infection results in an increase in BM multipotent non-committed progenitors

**Fig 1:**
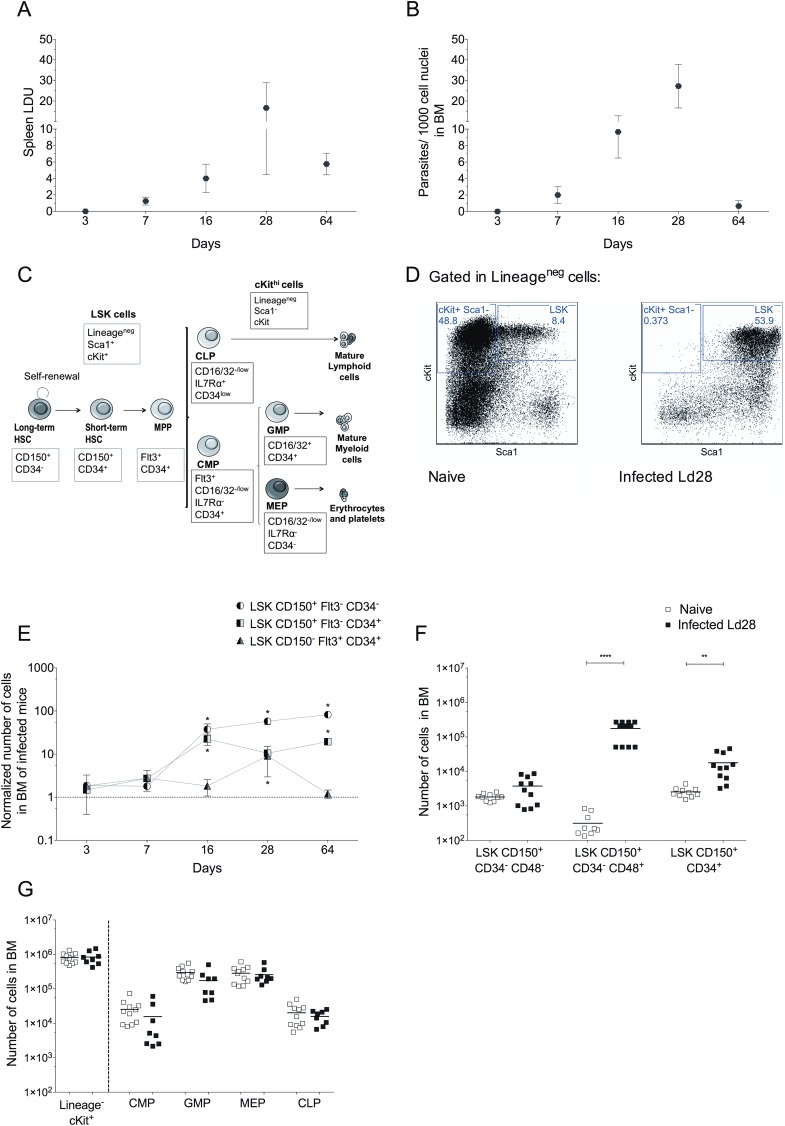
*L*. *donovani* infection increases the number of non-committed multipotent progenitors in BM. (A) Spleen parasite burden (LDU). (B) BM parasite burden. (C) Diagram of stepwise differentiation of hematopoietic precursors and panel of associated surface molecular markers used to characterize the different hematopoietic stem and progenitor cells (HSPCs). (D) Representative dot plots showing Sca1 and cKit expression on BM lineage negative cells for naive (left) and d28-infected mice (right). (E) Relative number of LSK CD150^+^Flt3^-^ CD34^-^ cells, LSK CD150^+^Flt3^-^CD34^+^ cells and LSK CD150^-^Flt3^+^CD34^+^ cells (Mean ± SD, normalized to average number calculated in naïve mice for two femurs and two tibias). *p ≤ 0.05, **p ≤0.01, ***p ≤0.001, by one-way ANOVA and Dunnett's Multiple Comparison Test (n = 4). (F) Number of progenitors separated by CD48 expression in BM of naïve and d28-infected mice (Ld28) (G) Number of lineage committed progenitors, as in (F). Three independent experiments (n = 8–12); Data presented as scatter plot and mean bar; *p ≤ 0.05, **p ≤0.01, ***p ≤0.001, ****p ≤0.0001 by unpaired t test.

We first characterised the impact of *L*. *donovani* infection (**Fig 1A and 1B**) on BM HSPCs using a panel of markers [8] (**S1 Table; Fig 1C**). In this analysis, we allowed for the finding that Sca1 is up-regulated on all progenitors after infection [6, 24], leading to a deficiency in the cKit^+^ Sca1^-^ and cKit^-^ Sca1^-^ cell populations compared to naïve mice (**Fig 1D**). Infection resulted in a significant increase in the number of multipotent Lineage^-^ cKit^+^ Sca1^+^ precursors in the BM that mirrored the course of infection and peaked on d28 post infection (p.i.) (**Fig 1E**). CD48 has been associated with a loss of stemness amongst LT-HSC [25–28]. Hence, we further characterised LSK CD150^+^CD34^-^ cells on the basis of CD48 expression. The number of LSK CD150^+^CD34^-^CD48^-^ cells (enriched for LT-HSCs) was unaltered in d28-infected mice compared to uninfected mice. In contrast, the numbers of LSK CD150^+^CD34^-^CD48^+^ cells and LSK CD150^+^CD34^+^ cells were significantly increased (**Fig 1F**). Notably, this increase in non-committed progenitors was not matched by increased numbers of lineage-committed precursors (**Fig 1G**), suggesting the possibility that HSPC differentiation was inhibited. Collectively, these findings indicate that infection had induced changes in hematopoietic differentiation prior to lineage commitment.

### Depletion of quiescent HSCs is a consequence of *L*. *donovani* infection

To evaluate whether *L*. *donovani* infection affected the function of HSPCs, we used a competitive adoptive transfer model. We selected this approach because it allows for the evaluation of the function of progenitor cells derived from infected and non-infected hosts in the same environment. Although long-term in vitro cultures are able to quantify more primitive progenitors, the growth factors required for LT-HSCs and their immediate progeny are not well established and may, therefore, impact the differentiation process [29].

**Fig 2:**
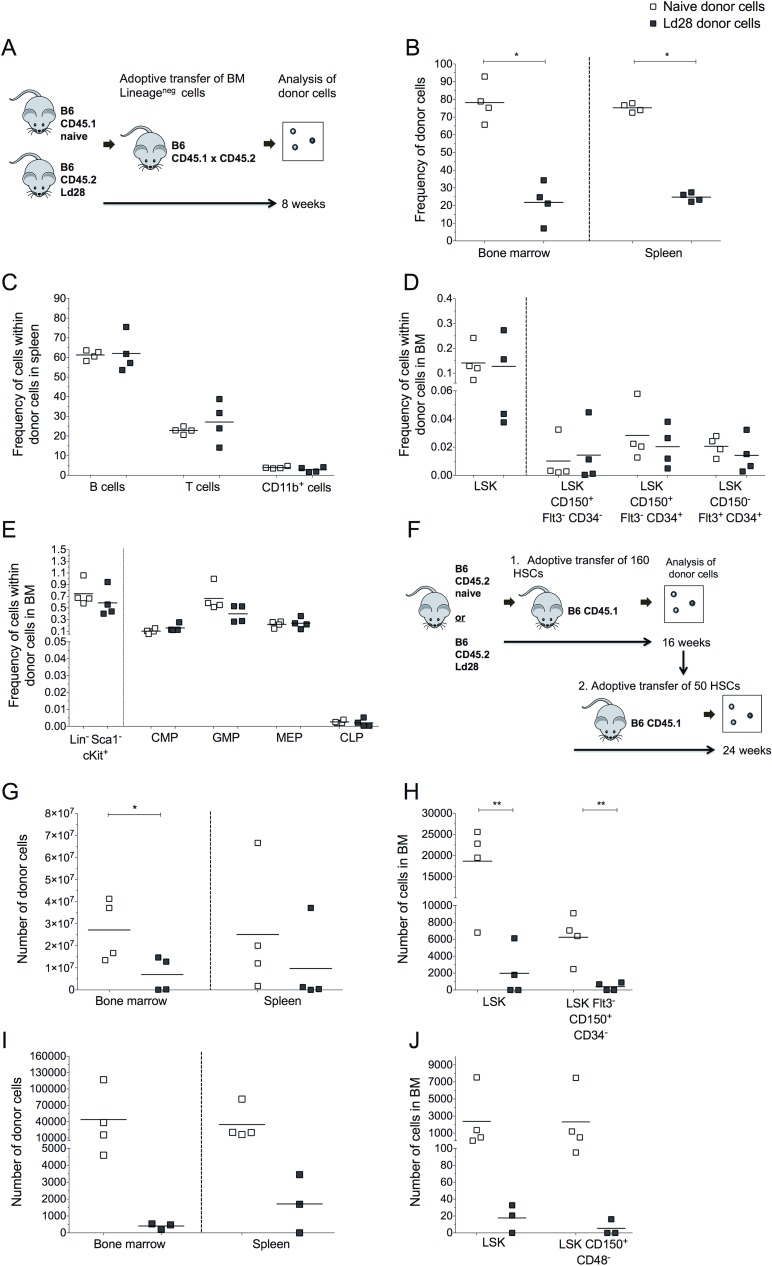
HSPCs from infected mice have defective capacity for self-renewal. (A) Diagram of experimental layout: naïve lethally irradiated B6.CD45.1xCD45.2 (n = 4) recipients received a 50:50 mix of BM Lin^-^ cells from d28-infected B6.CD45.2 and naïve B6.CD45.1 mice (A-D); (B) Frequency of donor hematopoietic cells in BM and spleen of recipient mice at 12 weeks post BMT; (C) Frequencies of donor leucocytes in spleen; (D) Frequencies of donor non-committed hematopoietic progenitor cells in BM; (E) Frequencies of lineage-committed progenitors in BM derived from each donor. (F) Diagram of experimental layout: naïve lethally irradiated B6.CD45.1 recipients received a radioprotective dose of 3x10^5^ CD45.1 cells along with 160 HSCs isolated from naïve or d28 infected B6.CD45.2 mice (F-H). (G) Number of donor hematopoietic cells in the BM and in the spleen of recipient mice at 16 weeks post BMT; (H) Number of donor non-committed multipotent progenitors cells in the BM of recipient mice. (I-J) Naïve irradiated B6.CD45.1 recipients received a radio protective dose of 3x10^5^ CD45.1 cells along with 50 HSCs of each origin derived from recipients (n = 3–4 per group) as shown in (F); (I) Number of donor hematopoietic cells in the BM and spleen of recipient mice at 24 weeks post BMT; (J) Number of donor non-committed multipotent progenitors cells in BM. Data presented as scatter plot and mean bar; *p ≤ 0.05, **p ≤0.01, ***p ≤0.001, ****p ≤0.0001 by unpaired t test.

BM lineage negative cells (enriched for HSPCs) from day 28 infected B6.CD45.2 mice and from uninfected B6.CD45.1 mice were mixed 50:50 and transferred into non-infected x-irradiated (B6.CD45.1 x B6.CD45.2)_F1_ recipients and cellularity assessed seven weeks later (**Fig 2A; S1A Fig**). HSPCs from infected mice contributed 21.81% ± 11.27 total donor cells in the BM of recipient mice and 24.76% ± 2.43 of total splenocytes (**Fig 2B**). Hence, HSPCs from infected mice have reduced competitiveness compared to HSPCs derived from naïve mice. No significant differences were noted in the frequency of B cells, T cells and CD11b^+^ myeloid cells, however, indicating that lack of HSPCs competitiveness was not associated with any evident lineage bias (**Fig 2C**). This was reflected in the similar frequencies of BM multipotent progenitors (**Fig 2D**) and lineage committed precursors (**Fig 2E**) derived from HSPCs from infected and naive mice. To determine whether LT-HSC might be infected with *L*. *donovani*, we infected mice for 28 days with Td-Tomato transgenic *L*. *donovani* and examined BM cells for the presence of amastigotes by flow cytometry. Although we observed that a very small percentage of BM lineage negative cells were infected with *L*. *donovani*, we did not observe infection of LT-HSCs (**S1E Fig**), ruling out infection of LT-HSCs as a reason for their altered competitiveness. These data suggested that infection results in HSC-intrinsic functional impairment that occurs prior to lineage commitment.

To test this hypothesis, we initially performed a long-term non-competitive adoptive transfer experiment, placing LSK CD150^+^ CD34^-^ CD48^-^ cells (LT-HSCs) from CD45.2 naive or infected mice into naive CD45.1 recipients (**Fig 2F**). Donor LT-HSCs from infected mice showed a trend towards poorer reconstitution in BM (2.72 x 10^7^ ± 1.41 x 10^7^ cells vs. 6.96 x 10^6^ ± 7.89 x 10^6^ cells, naive vs. infected mice), and the same was observed in the spleen (**Fig 2G**). We could not detect any significant alteration in the distribution of mature spleen cells derived from infected compared to naive donor cells, again suggesting no lineage bias arises from HSCs from infected mice (**S1B Fig**). However, the number of LSK cells and LSK CD150^+^ FLT3^-^ CD34^-^ cells (enriched in LT-HSCs) derived from infected donors was significantly decreased compared to naïve donors (**Fig 2H**), again indicating that HSCs from infected mice were less able to reconstitute the hematopoietic system and their self-renewal potential was compromised.

To further investigate the long-term reconstituting potential and to more accurately evaluate the reduction in functional capacity of HSCs from infected mice, we transferred 50 LSK CD150^+^ CD34^-^ CD48^-^ cells that were isolated from the primary adoptive transfer recipients into secondary recipients (**Fig 2F**). We identified BM and spleen cells in all (4/4) recipients of LT-HSCs originally obtained from naive mice. In contrast, only 2/3 BM and 2/3 spleens examined had detectable cells derived from HSCs originally taken from infected mice and these were very rare in number (**Fig 2I**). In all secondary recipient mice transplanted with LT-HSCs isolated from non-infected mice, we could detect donor HSPCs for the three lineages in the BM of recipient mice and, following an overall period of 40 weeks of transfer into healthy recipients, LT-HSCs derived from naive donors were capable of self-renewal and of giving rise to all lineages. In contrast, we only found LSK CD150^+^ CD48^-^ cells derived from infected donors in 1/3 recipients and we failed to find lineage-committed progenitors or mature progeny in 2/3 recipients (**Fig 2J**, **S1C and S1D Fig**). Collectively, these data suggested that HSCs from infected mice show intrinsic long-term functional impairment.

**Fig 3:**
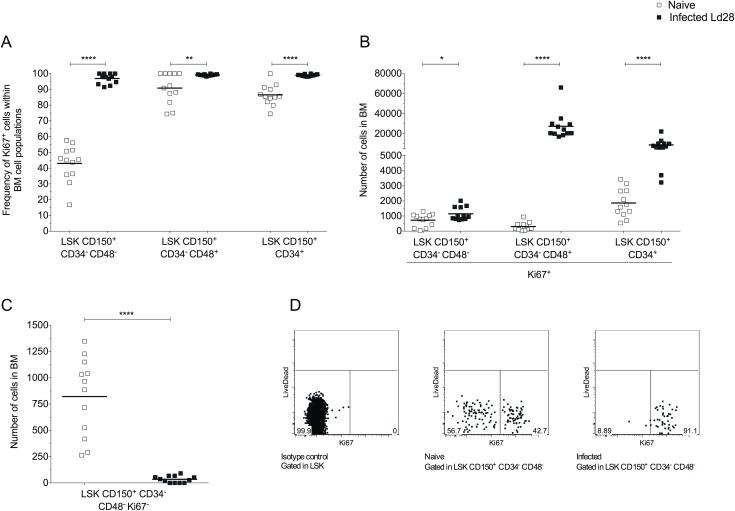
Loss of quiescent LT-HSCs following *L*. *donovani* infection. (A and B) Frequency (A) and number (B) of Ki67^+^ within HSPCs populations in BM of naïve and d28-infected B6 mice. (C) Number of LT-HSCs in G0 (Ki67^-^) in BM in naïve and d28-infected mice (n = 12 per group; three independent experiments). Data presented as scatter plot and mean bar; unpaired t test; *p ≤ 0.05, **p ≤0.01 ****p ≤0.0001. (D) Representative dot plots for Ki67 expression on LSK CD150^+^CD34^-^CD48^-^ cells.

In naïve mice, LT-HSCs are commonly found in G0 (Ki67^-^), and a strict regulation of LT-HSC proliferative status is required for the maintenance of long-term self-renewal and multi-lineage differentiation potential [30, 31]. We found that approximately 40% of LT-HSCs in naïve mice expressed Ki67 (**Fig 3A** and **3D**). At day 28 p.i., however, the frequency of LT-HSCs in cell-cycle was significantly increased (96.52% ± 3.19) (**Fig 3A** and **3D**). Onward populations of non-committed progenitors were highly proliferative both in naïve and infected mice, and following infection these resulted in an increased number of LSK CD150^+^ CD34^-^ CD48^+^ Ki67^+^ cells and LSK CD150^+^ CD34^+^ Ki67^+^ cells in infected mice (90-fold and 4.8-fold, respectively, compared to naive mice) (**Fig 3Ai and 3B**). Furthermore, the loss of LSK CD150^+^ CD34^-^ CD48^-^ Ki67^-^ cells during infection represented a 24-fold decrease in number (from 831 ± 0.017 to only 34.62 ± 31.93 cells in total BM; **Fig 3C**). Hence, depletion of the reservoir of LT-HSCs due to proliferation may account for poor reconstitution efficiency shown by HSPCs from infected mice.

To determine the mechanisms regulating proliferation, we assessed the expression of the transcription factor GATA-3, as recent studies have suggested that GATA-3 is important in the regulation of HSC proliferative status and self-renewal potential during stress-induced hematopoiesis [32, 33]. We observed an infection-associated increase in GATA-3 expression in most immature cells (**S2A Fig**). We next determined whether the expression of GATA-3 could be related to the proliferative state of LSK CD150^+^ CD48^-^ cells. Few cells in G0 expressed GATA-3 but concomitant with infection, we observed a significant increase in LSK CD150^+^ CD48^-^ cells expressing Ki67 and GATA-3 (17.64 ± 11.23 vs. 46.68 ± 21.66, in naive vs. infected mice, respectively) (**S2B Fig**), and in infected mice we determined a significant alteration in the distribution of LSK CD150^+^ CD48^-^ cells segregated according to the expression of GATA-3 and Ki67 (**S2B Fig**). As such, GATA-3 over expression due to inflammation may represent a mechanism for impaired maintenance of homeostatic numbers of quiescent LT-HSCs during *L*. *donovani* chronic infection.

### Accumulation of “effector” BM CD4^+^ T cells is associated with altered LT-HSC function

**Fig 4:**
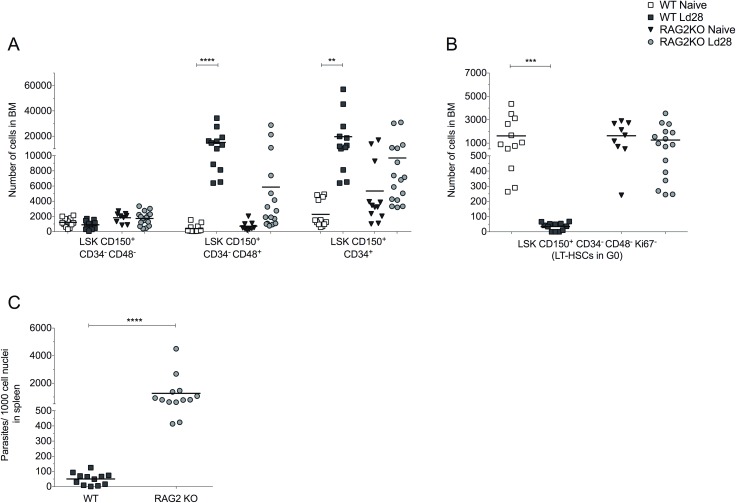
Quiescent LT-HSCs are retained in infected *Rag2*^-/-^ mice. (A) Number of HSPCs in BM of naïve B6.WT (light squares), naïve B6.*Rag2*^-/-^ (dark grey triangle), d28 infected B6 (dark grey squares) and d28 *Rag2*^-/-^ (light grey circles) mice. (B) Number of BM LT-HSCs in G0 (Ki67^-^). (C) Spleen parasite burden in B6.WT and B6 *Rag2*^-/-^, presented as number of parasites per 1000 nuclei. Data from three independent experiments presented as scatter plot and mean bar (n = 9–16 per group); *p ≤ 0.05, **p ≤0.01, ***p ≤0.001 and ****p ≤0.0001; unpaired t test.

In contrast to wild type (WT) B6 mice, immunodeficient Recombination activating gene 2 (*Rag2*) knockout mice showed no signs of infection-associated changes in HSPCs, despite a significantly higher parasite burden (Fig **4A** and **4C**). Furthermore, infection of *Rag2* KO mice did not deplete the reservoir of quiescent HSCs (**Fig 4B**), suggesting a central role for adaptive immunity as a driver of these effects in immunocompetent mice.

**Fig 5:**
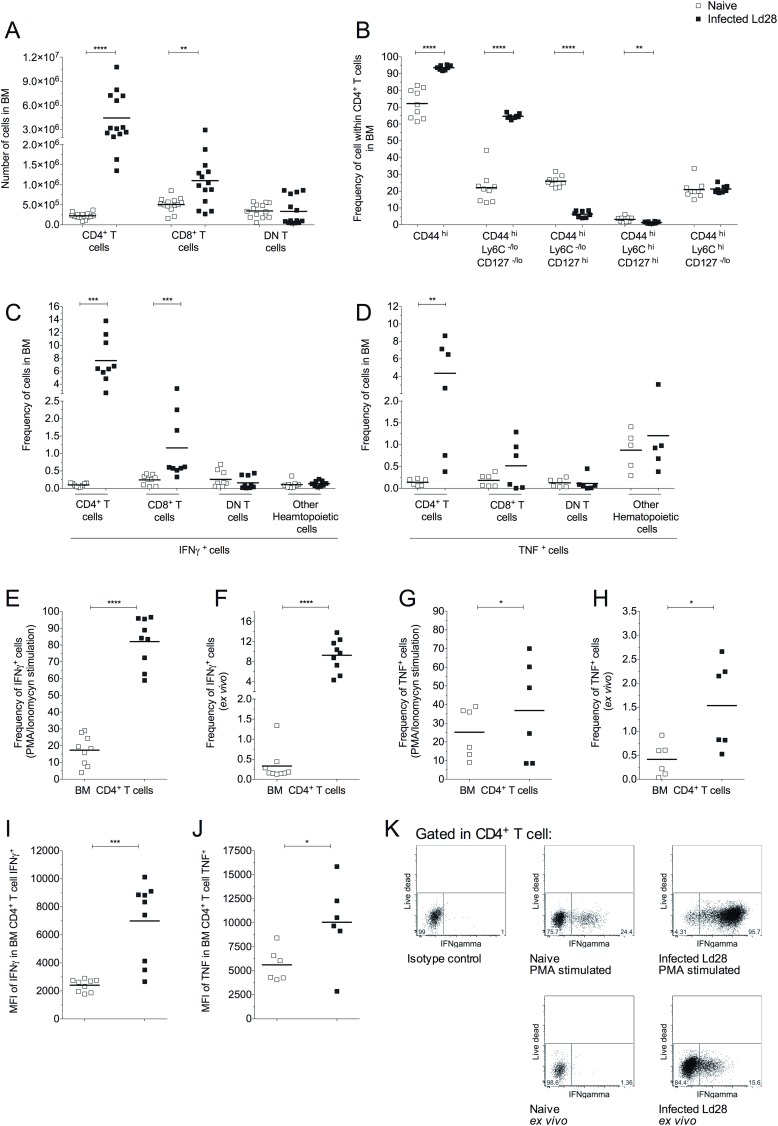
*L*. *donovani* infection expands the population of BM T cells expressing IFNγ and TNF. Comparison of BM T cells in naïve and d28-infected mice (Ld28). (A) Number of T cells in BM. (B) Frequency of total CD44^high^ and CD44^high^ subsets within total BM CD4^+^ T cell population. (C) Frequency of IFNγ^+^ subsets in total BM cells. (D) Frequency of TNF^+^ subsets in total BM cells. (E) Frequency of IFNγ^+^ within BM CD4^+^ T cell population following stimulation *in vitro*. (F) Frequency of IFNγ^+^ within BM CD4^+^ T cell population directly *ex vivo*. (G) Frequency of TNF^+^ within BM CD4^+^ T cell population following stimulation *in vitro*. (H) Frequency of TNF^+^ within BM CD4^+^ T cell population directly ex vivo. (I) Mean Fluorescence Intensity (MFI) of IFNγ in IFNγ^+^CD4^+^ T cells. (J) MFI of TNF in TNF^+^CD4^+^ T cells. Data from at least two independent experiments (n = 6–14 per group) presented as scatter plot and mean bar; *p ≤ 0.05, **p ≤0.01, ***p ≤0.001 and ****p ≤0.0001; unpaired t test. (K) Representative dot plots for IFNγ expression by stimulated BM CD4^+^ T cells (top) or *ex vivo* (bottom).

In WT mice, we found that BM CD4^+^ T cells increased in number 24-fold on infection, whereas CD8^+^ T cells increased ~2-fold (**Fig 5A**). To determine if these BM CD4^+^ T cells were phenotypically similar to the recently described BM resident effector cells, we examined the expression of CD44, CD127 and Ly6C [1, 34]. In the BM, a majority of CD4^+^ T cells were CD44^high^ cells, increasing from 72.22% ± 8.67 of total CD4^+^ T cells in naive mice to 93.56% ± 1.39 in infected mice. The most abundant population of CD4^+^ T cells was CD44^high^ Ly6C^-/low^ CD127^-/low^ (“effector T cells”) (**Fig 5B**), consistent with the accumulation of effector CD4^+^ T cell in BM during infection with *L*. *donovani*.

To characterize their potential for cytokine production, we used *in vitro* stimulation with PMA and ionomycin followed by flow cytometry (**Fig 5C–5K**). The frequency of BM CD4^+^ T cells capable of producing IFNγ within the total BM was very low (0.10% ± 0.05) in naive mice, but increased following infection (7.64% ± 3.57 of total BM cells; **Fig 5C**). The percentage of CD8^+^ T cells with the potential to produce IFNγ also increased in the BM of infected mice but to a lesser extent (0.24% ± 0.14 vs 1.16% ± 1.03, naive vs. infected), and there was no indication of other relevant sources of IFNγ (**Fig 5C**). Within the CD4^+^ T cell population of infected mice, 82.06% ± 14.23 of cells had the potential to produce IFNγ compared to 17.33% ± 8.95 in naive controls (**Fig 5E and 5K**), and a significant increase in the frequency of cells expressing IFNγ driven by *L*. *donovani* infection was also determined directly *ex vivo* (**Fig 5F and 5K**). Analysis of the MFI for INFγ^+^ within CD4^+^ T cells also demonstrated an increase in cytokine production on a per cell basis, compared to CD4^+^ T cells from naive mice (**Fig 5I**). Similar data were also obtained for TNF production by BM CD4^+^ T cells (**Fig 5D, 5G, 5H and 5J**). Overall these data show that *L*. *donovani* infection stimulated marked recruitment / expansion of highly active BM effector CD4^+^ T cells.

### CD4^+^ T cells mediate LT-HSC exhaustion via IFNγ-dependent mechanisms

**Fig 6:**
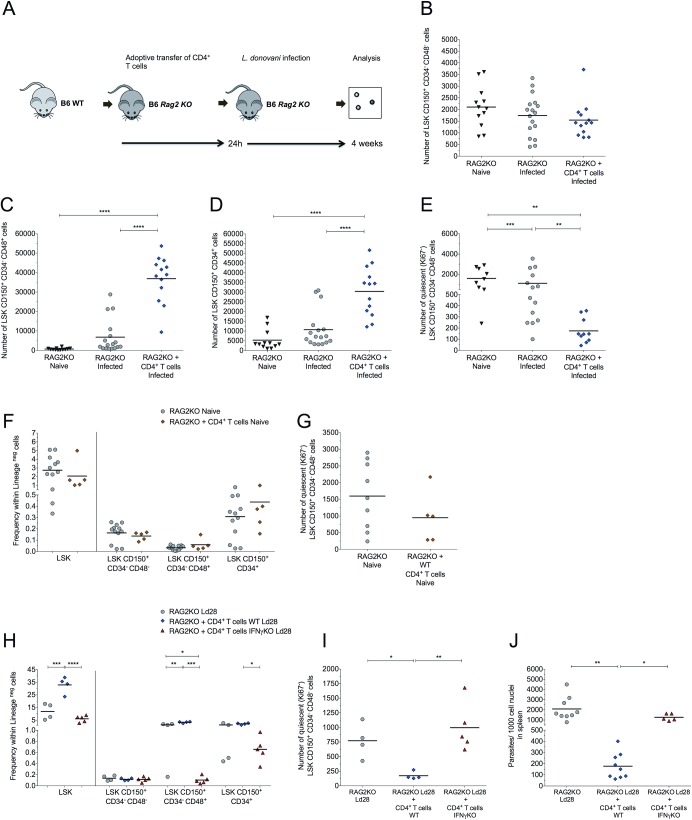
CD4^+^ T cells drive HSC exhaustion in an IFNγ-dependent manner. (A) Diagram of experimental layout, RAG2 KO mice were adoptively transferred with sorted CD4^+^ T cells from naive mice, and then infected in the following day with *L*. *donovani*. At day 28 p.i., we analysed the distribution of hematopoietic progenitors in the BM in naïve RAG mice, infected RAG2 KO mice and infected RAG mice that receive adoptively transferred CD4^+^ T cells: (B-E) Number of HSPCs: LT-HSCs (B), LSK CD150^+^CD34^-^CD48^+^ cells (C), LSK CD150^+^CD34^+^ cells (D) and quiescent LT-HSCs LSK in BM (E) (n = 12–17 per group, from three independent experiments). (F) Frequency of HSPCs populations within Lineage negative cells in naïve RAG mice with and without adoptive CD4^+^ T cell transfer; and (F) number of quiescent LT-HSCs (n = 9–5 per group). (H-J) Frequency of progenitor cells within Lineage negative cells in infected RAG mice without or with adoptive transfer of IFNγ sufficient or IFNγ-deficient CD4^+^ T cells (G); Number of quiescent LT-HSCs (n = 4–5) (H). (J) Parasites per 1000 nuclei in the spleen. Data presented as presented as scatter plot and mean bar; *p ≤ 0.05, **p ≤0.01, ***p ≤0.001 and ****p ≤0.0001; one-way Anova and Tukey’s multiple comparisons test.

We hypothesized that BM CD4^+^ T cells might drive the alterations observed in the hematopoietic compartment during infection. To assess whether this was the case, *Rag2* KO mice were adoptively transferred with sorted CD4^+^ T cells from naive B6 mice, and then infected with *L*. *donovani* (**Fig 6A**). The number of LT-HSCs was unchanged in all three groups (**Fig 6B**), whereas the number of intermediary non-committed progenitors (LSK CD150^+^CD34^-^CD48^+^ cells and LSK CD150^+^CD34^+^ cells) increased in adoptively transferred *Rag2* KO mice to a similar extent as observed in infected WT mice (**Fig 6C and 6D**). More importantly, we found that whereas infected *Rag2* KO mice preserved their reservoir of quiescent LT-HSCs, this was reversed following CD4^+^ T cell transfer (**Fig 6E**). In contrast, adoptively transferred *Rag2* KO mice that were not infected retained their full quiescent HSPC pool, indicating that infection rather than homeostatic expansion of CD4^+^ T cells is required to drive LT-HSCs out of quiescence (**Fig 6F and 6G**). As IFNγ signalling has been associated with altered HSC function [6, 7, 10, 11, 35], we assessed the competency of IFNγ^-/-^ CD4^+^ T cells to regulate loss of quiescence in infected *Rag2* KO recipients. All effects attributed to the transfer of CD4^+^ T cells to *Rag2* KO recipients described above were lost when these cells were incapable of producing IFNγ (**Fig 6H and 6I**), defining CD4^+^ T cell-derived IFNγ as a critical regulator of LT-HSC function during *L*. *donovani* infection. On the other hand, IFNγ was critical to control parasite burden (**Fig 6J**), indicating that the mechanisms underlying host resistance to infection may also impact hematopoietic function when sustained over time.

### Intrinsic IFNγR signalling in HSCs is not required for loss of quiescence

**Fig 7:**
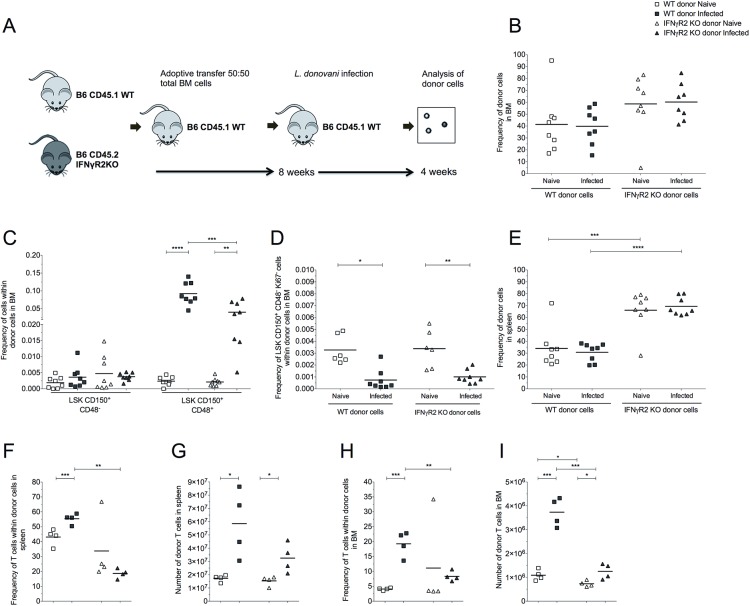
Intrinsic IFNγ receptor signaling is required for expansion of BM T cells following infection. (A) Experimental design for competitive mixed BM chimeras using wild-type (WT) and *Ifnγr2* knockout (IFNγR2 KO). Analyses were performed 12 weeks after BMT from CD45.2 *Ifnr2*^-/-^ mice and CD45.1 WT mice (50:50) to lethally irradiated CD45.1 recipient mice, subsequently infected with *L*. *donovani* for 28 days. (B) Frequency of donor cells in BM. (C) Frequency of BM LSK CD150^+^ CD48^-^ cells (enriched for LT-HSCs) and LSK CD150^+^ CD48^+^ cells within donor cells. (D) Frequency of LT-HSCs in G0 (Ki67^-^) within donor cells. (E) Frequency of donor cells in spleen. (F) Frequency of T cells within donor cells in spleen. (G) Number of donor T cells in spleen. (H) Frequency of BM T cells within donor cells. (I) Number of donor T cells in the BM. Data presented as scatter plot and mean bar (n = 4–8); *p ≤ 0.05, **p ≤0.01, ***p ≤0.001 and ****p ≤0.0001; One-way Anova followed by Tukey’s multiple comparisons test.

HSCs express receptors for IFNγ, which have been directly associated with LSK expansion and impaired engraftment in X-irradiated hosts [7, 35]. To test whether LT-HSC intrinsic IFNγR signalling was required for loss of quiescence, we generated 50:50 mixed BM chimeras using cells derived from both WT and *Ifnγr2*^-/-^mice (B6.CD45.1 + B6.CD45.2.*Ifnγr2*^-/-^→B6.CD45.1) (**Fig 7A**). The percentage of BM cells derived from *Ifnγr2*^-/-^ donor cells was comparable to WT donor cells (**Fig 7B**). In infected recipient mice, the frequency of WT and *Ifnγr2*^-/-^ LSK CD150^+^ CD48^-^ cells in the BM was similar, as was the increase in frequency of LSK CD150^+^ CD48^+^ cells derived from WT donor cells and *Ifnγr2*^-/-^ donor cells (**Fig 7C**). Most importantly, loss of quiescent HSCs was observed equally for WT and *Ifnγr2*^-/-^ derived cells (**Fig 7D**). Thus, intrinsic IFNγ signalling was not mediating the expansion of multipotent progenitors or LT-HSC exhaustion in this model.

Nonetheless, significant changes in the frequency of myeloid progenitors and mature myeloid cells derived from WT and *Ifnγr2*^-/-^ were observed following infection, suggesting that IFNγ signalling may modulate the generation of myeloid cells in response to *L*. *donovani* infection (**S3A, S3C and S3E Fig**). There was no indication that IFNγ signalling played a major role regulating the B cell compartment following infection (**S3B and S3D Fig**).

Lack of IFNγR2 led to an increase in the frequency of donor cells in the spleen compared to WT donor cells, suggesting that IFNγ signalling impacts on hematopoietic function both in steady-state and under inflammatory conditions (**Fig 7E**). In BM and spleen of non-infected recipient mice, the frequency and absolute number of T cells was comparable between WT and *Ifnγr2*^-/-^ donor cells, indicating that IFNγ signalling in T cells was not required for their development or homeostatic maintenance. In contrast, the lack of IFNγR2 intrinsic signalling prevented the expansion of the T cell compartment following infection that was seen with WT T cells, both in BM and in the spleen (**Fig 7F–7I**). As such, intrinsic IFNγ signalling confers a proliferative or survival advantage for CD4^+^ T cells during *L*. *donovani* induced inflammation, but not under conditions of homeostatic expansion.

### Intrinsic TNFR signalling modulates expansion of BM CD4^+^ T cells

**Fig 8:**
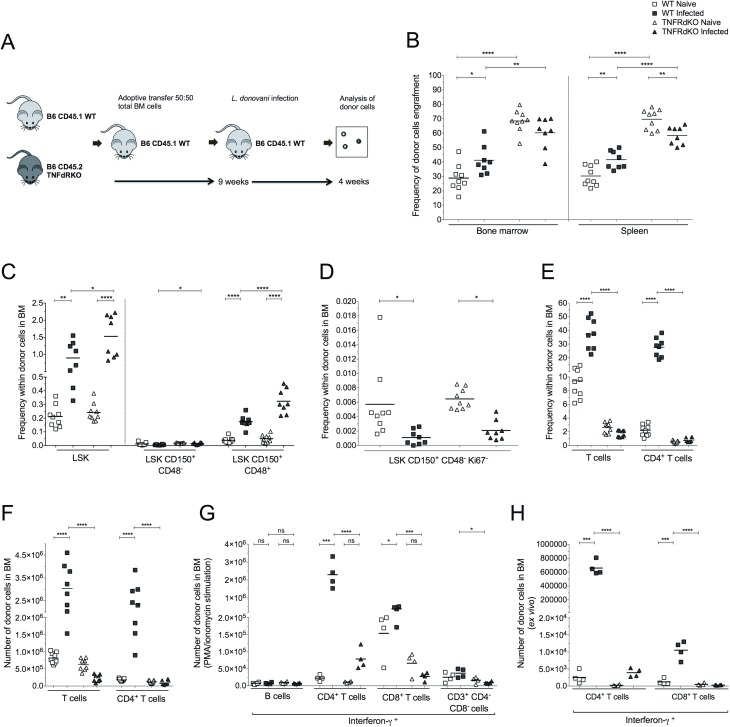
T cell intrinsic TNF receptor signaling is required the expansion of pathogenic CD4^+^ T cells following infection with *L*. *donovani*. (A) Experimental design for competitive mixed BM chimeras using wild-type (WT) and *Tnfrsf1*-dKO (TNFdRKO), subsequently infected with *L*. *donovani* for 28 days. Analyses were performed 13 weeks after BMT from CD45.2 TNF-RdKO mice and CD45.1 WT mice (50:50) to lethally irradiated CD45.1 recipient mice. (B) Frequency of donor cells in BM and spleen. (C) Frequency of HSPCs within donor cells in BM. (D) Frequency of BM LSK CD150^+^ CD48^-^ cells (enriched for LT-HSCs) in G0 (Ki67^-^) within donor cells. (E) Frequency of total BM T cells and BM CD4^+^ T cells within donor cells. (F) Number of T cells and CD4^+^ T cells in BM. (G) Number of BM leucocytes expressing IFNγ in naïve and infected mice derived from B6 or *Tnfrsf1*-dKO donor HSCs following PMA/ionomycin stimulation i*n vitro*. (H) Number of BM T cells and BM CD4^+^ T cells IFNγ^+^ cells derived from B6 or *Tnfrsf1*-dKO donor HSCs measured directly *ex vivo*. *p ≤ 0.05, **p ≤0.01, ***p ≤0.001 and ****p ≤0.0001; One-way Anova followed by Tukey’s multiple comparisons test.

TNF has also been proposed to play an important role in directly modulating HSC function and may cooperate with other mechanisms in driving stress-induced hematopoiesis and mediating hematopoietic dysfunction [36]. Non-committed progenitors upregulated the expression of receptors for TNF (TNFR1a and TNFR1b) during *L*. *donovani* infection (**S4 Fig**). To formally test whether intrinsic TNF signalling plays a role in driving LT-HSCs into active cell-cycle and subsequent LT-HSC exhaustion, we transferred equal numbers of BM cells from WT and *Tnfrsf1*-dKO donors into lethally irradiated recipients **(Fig 8A)**. In naïve chimeras assayed at 13 weeks post BM transfer (BMT), *Tnfrsf1*-dKO cells showed a clear competitive advantage (representing 68.51% ± 7.58 of total cells in BM; **Fig 8B**). This was also observed in the spleen and suggested that TNF signalling may modulate hematopoietic function during homeostasis. Although minor changes in relative frequency occurred following infection, the bias towards *Tnfrsf1*-dKO cells was not enhanced (**Fig 8B**). Loss of both TNFRs had no impact on the expansion of LSK cells (**Fig 8C**) or on the loss of quiescent LT-HSCs (**Fig 8D**). Minor changes were observed in the lineage-committed progenitors, B cells and CD11b^+^ cells in the BM and spleen of infected recipients (**S5A, S5B, S5C and S5D Fig**). These data argue against a role for LT-HSC intrinsic TNF signalling in the expansion of multipotent progenitors and in LT-HSC exhaustion.

Remarkably, in non-infected chimeric mice, the frequencies and number of total BM T cells and BM CD4^+^ T cell derived from WT and *Tnfrsf1*-dKO donor cells were similar, whereas following infection differences in T cells derived from WT and *Tnfrsf1*-dKO donor cells became evident in the BM. *Tnfrsf1*-dKO CD4^+^ T cells did not increase in frequency or number following infection, whereas WT CD4^+^ T cells expanded approximately 15-fold (**Fig 8E and 8F**). Likewise, in the spleen WT T cells but not *Tnfrsf1*-dKO T cells increased in frequency and number, although to a lesser extent than in the BM (**S5E and S5F Fig**). Thus, TNF acts directly on BM CD4^+^ T cells to regulate their expansion following infection.

### Loss of TNFR signalling prevents expansion of IFNγ-producing BM CD4^+^ T cells

As the expansion of BM CD4^+^ T cells following infection was prevented in cells lacking TNF signalling receptors, we used the mixed chimeras described above to assess whether T cells devoid for TNF signalling were impaired in their efficiency to produce IFNγ, a cytokine critical in regulating LT-HSC exhaustion (**Fig 6**). In the BM of these infected mice, 2.29x 10^6^ ± 7.67x10^5^ WT CD4^+^ T cells had the potential to express IFNγ compared to 7.87x10^4^ ± 3.07x10^4^ *Tnfr1*/*Tnfr2* double KO donor cells (**Fig 8G**). This disparity between WT and *Tnfr1*/*Tnfr2* double KO donor CD4^+^ T cells was further amplified in terms of absolute number of IFNγ producing cells analysed directly *ex vivo* (**Fig 8H**). TNFR deficiency also impacted on the ability of CD8^+^ T cells to produce IFNγ, although their contribution to the overall level of this cytokine in BM was minor (**Fig 8G and 8H**). Collectively these data demonstrate that TNF acting directly on CD4^+^ T cells is required for the accumulation of a pathogenic population of CD4^+^ T cells expressing IFNγ in BM, which in turn mediates exhaustion of HSCs in mice infected with *L*. *donovani*.

## Discussion

Although it is well established that impairment of hematological function occurs during VL, the underlying mechanisms are poorly understood [15]. In the current study, we demonstrate that CD4^+^ IFNγ^+^ effector T cells expand in the BM of *L*. *donovani* infected mice and drive LT-HSCs into a state of functional exhaustion. Our data define a new pathogenic role of CD4^+^ T cells in this disease model, and describe a novel TNF signalling-dependent pathway for regulating effector T cells that may also have relevance for other diseases characterised by hematological dysfunction.

In the current study, we have focused on early events in hematopoiesis examining the fate and self-renewal potential of LT-HSCs in mice. We have identified for the first time that following *L*. *donovani* infection early hematopoietic progenitors accumulate in BM due to an increase in active proliferation, but at the expense of the reservoir of quiescent LT-HSCs. We found that alteration in the proliferative status of LT-HSCs was associated with the upregulation of the expression of GATA-3, a transcription factor previously associated with loss of reconstitution potential of HSCs [37], and confirmed loss of self -renewal capacity through serial transfer. As residual HSC function was reflected by multilineage differentiation, within the constraints of our analysis, the impact of infection with *L*. *donovani* does not appear to extend to the epigenetic effects that have been reported in aging and some hematological malignancies [38].

Our findings are in agreement with and extend previous studies that have reported alterations in the proliferative status and self-renewal capacity of LT-HSCs under pro-inflammatory conditions [10, 11, 33, 35, 39]. Similar alterations in HSC behaviour have also been described in other models of infection although a consensus on whether this is host beneficial or detrimental has not been reached [7, 24, 40, 41]. Unlike here, previous studies have not provided a causal link between changes in cytokine profile, T cell response and HSC exhaustion. A role for T cells has, however, been mooted. Using experimental models for aplastic anemia, T cells were proposed as key mediators of hematopoietic dysfunction and ultimately BM failure [4, 5], and in experimental ehrlichiosis, infection-induced expansion of LSK cells was shown to be dependent upon IFNγ production by CD4^+^ T cells [7].

We found clear alterations in the composition of T cells residing in the BM following infection, notably a dramatic increase in the frequency and number of CD4^+^ T cells displaying an “effector” phenotype and secreting high levels of IFNγ. Antigen-specific CD4^+^ T cells producing IFNγ are a well characterised feature of VL, both in murine models and in humans and play an important role in immune protection [42–44]. However, this is the first report addressing the profile of cytokine expression in BM CD4^+^ T cells following experimental *L*. *donovani* infection. In adoptive transfer settings, the production of IFNγ by CD4^+^ T cells was sufficient to drive LT-HSC exhaustion defining this pro-inflammatory cytokine as a key regulator of hematopoiesis during infection with *L*. *donovani*. Strikingly, the absence of CD4^+^ T cell-intrinsic TNF signalling prevented their expansion in the BM of infected mice, and limited their potential to produce IFNγ, indicating that TNF plays a central upstream role in regulating the BM T cell compartment during infection. Although evidence of T cell function in the BM of patients with VL is scare, available data also suggests that IFNγ is more abundant in BM aspirate fluid than in serum in active VL patients and that higher than baseline levels of IFNγ persist in BM post cure [45].

Alterations in the proliferative status of HSCs and their subsequent functional impairment have been linked to IFNγ in other situations of stress-induced hematopoiesis, and it has been suggested that this reflects the effects of direct HSC-intrinsic IFNγR signalling [10, 11, 46]. Thus, IFNγR-signalling on HSCs has been viewed as a pivotal mechanism to explain alterations in HSC function under inflammatory conditions [47–50]. Likewise, a similar role was more recently suggested for HSC-intrinsic TNFR signalling during stress-induced hematopoiesis [36]. To test the hypothesis that IFNγ signalling and/or TNFR signalling in LT-HSCs were driving increased proliferation during infection with *L*. *donovani*, we established BM mixed chimeras with equal number of total BM cells derived from WT and IFNγR2KO or TNFRdKO. This approach allowed the experimental evaluation of the impact of IFNγR and TNFR signalling in LT-HSCs, whilst excluded the confounding effect of global loss of IFNγ or TNF signalling evident in previous studies [10, 11, 46]. Our findings obtained using a mixed BM chimeric model do not support these conclusions. Rather, our data clearly demonstrate that LT-HSCs in a cytokine replete environment are driven into exhaustion in an identical manner, irrespective of whether they express IFNγR2 or TNFR. Our findings strongly suggest that, in contrast to directly affecting LT-HSCs, IFNγand TNF receptor signalling converge at the level of the CD4^+^ T cells to regulate expansion of a highly-activated effector population. Further work will be required to establish the cellular target of IFNγ produced by BM CD4^+^ T cells in *L*. *donovani*-infected mice. For example, changes in stromal cell function due to exposure to IFNγ have been proposed to explain the increased myelopoiesis in mice infected with Lymphocytic choriomeningitis virus (LCMV) strain WE [51].

Intrinsic IFNγR signalling has been suggested to regulate T cell differentiation in other settings [52, 53]. For example, in mice immunized with LCMV, it was shown that antigen-specific CD4^+^ T cells expand at a much higher rate compared to CD4^+^ T cells lacking IFNγ signalling [53]. In contrast, in experimental *Listeria monocytogenes* infection, the expression of IFNγ by CD4^+^ T cells was negatively regulated by IFNγR [52]. Finally, in mixed BM chimeras infected with *E*. *chaffeensis*, IFNγR-deficient T cells were increased in frequency compared to IFNγR-sufficient T cells [6], contrary to our observations with *L*. *donovani* in similar chimeric mice. Together, these studies argue for the existence of potentially diverse mechanisms of immune control that are exaggerated under different infection conditions.

A key finding from our work is the identification of a role for intrinsic TNFR signalling in CD4^+^ T cells as a key step in their differentiation into cells able to drive LT-HSC exhaustion. Following *L*. *donovani* infection the lack of intrinsic TNF signalling led to a 30-fold reduction in the number of BM CD4^+^ cells expressing IFNγ. Therefore, TNF emerges as a crucial mediator in the development of this pathogenic population of CD4^+^ T cells in the BM, as well as in the spleen, of *L*. *donovani*-infected mice. As with studies on the role of IFNγR, the literature is divided over the role of intrinsic TNFR signalling in CD4^+^ T cells. For example, a lack of intrinsic TNF-R1b signalling has been reported to significantly curtail expansion of CD4^+^ T cells in response to low concentrations of specific antigen and compromise the ability of T cells to express IFNγ [54], whereas lack of TNF or TNFR signalling lead to uncontrolled expansion of IFNγ^+^ T cells following BCG infection [55, 56].

Compared with the spleen, T cells enriched for an “activated” phenotype reside in greater numbers in the BM of naive animals ([2, 57]) and persist for much longer periods of time in BM compared to other lymphoid tissues following infection resolution [2, 58]. These data, in combination with our findings suggest that the development of highly activated BM homing CD4^+^ T cells, induced not only by *Leishmania* infection, but by a variety of infectious challenges, may account for erosion of hematological function over time. Furthermore, the link established here between TNF and the development of BM CD4^+^ T cells with potential to irreversibly impact on LT-HSC function may be of importance in other non-infectious diseases where TNF production and hematological abnormalities co-exist.

In summary, we propose a mechanism (**S6 Fig**) whereby following infection: (i) IFNγ and TNF produced as part of the ongoing immune response co-operate at the level of receptor signalling on CD4^+^ T cells to promote accumulation of highly activated effector CD4^+^ IFNγ^+^ cells in the BM; (ii) in these cells, TNF signalling (possibly in association with other mediators) drive the expression of IFNγ; (iii) IFNγ produced by CD4^+^IFNγ^+^ T cells operates indirectly to cause LT-HSCs to enter active cell cycle; and (iv) chronic stimulation of LT-HSCs via this pathway leads to their exhaustion through loss of quiescence. Our model also suggests that intermediary progenitors which we show accumulate in BM during chronic infection may be less efficient at producing mature progeny (ineffective hematopoiesis), but we have not specifically addressed whether this reflects an intrinsic defect or a further consequence of residence in an environment chronically exposed to cytokines such as TNF and IFNγ. Recent studies by others have, for example, shown that stress-induced hematopoiesis promoted by chronic pro-inflammatory conditions results in DNA damage that impaired the differentiation of mature functional progeny [39].

Given the well-established role of CD4^+^ T cell-derived IFNγ in in the control of the parasite burden in VL [59, 60], that we confirmed in the present study, there is likely to be a trade-off between on the one hand the need for an effective anti-parasitic response to control primary infection versus on the other hand the potential for longer term and irreversible damage to hematopoietic fitness. The importance of this immunopathological sequela for the long-term health of patients faced with continued pathogen insult over their life course remains to be evaluated.

## Materials and methods

### Ethics statement

All animal experiments were carried out in accordance with the Animals and Scientific Procedures Act 1986, under UK Home Office Licence (project licence number PPL 60/4377 approved by the University of York Animal Welfare and Ethics Review Board), and conformed to ARRIVE guidelines. Animals were killed by CO_2_ asphyxia and cervical dislocation.

### Animal and infections

B6.CD45.1, B6.CD45.2, (B6.CD45.1xCD45.2)F1, B6.EYFP.*Rag1*^-/-^ and B6.*Rag2*^-/-^.CD45.1Cg were used in this study, bred and maintained under specific-pathogen free (SPF) conditions at the Biological Services Facility, University of York. BM cells from mice lacking the *Ifngr2* gene (IFNγ-R2 KO) on a B6 background were generously provided by Dr. Grainger (University of Manchester, UK) [61]. BM cells from TNF receptor double KO mice (Tnfrsf1-dKO B6.129S mice) [62] backcrossed >10 generations to C57BL/6 mice were provided by Dr. Lindbom (Lund University, Sweden). IFNγ-KO (B6.129S7-*Ifng*tm1Ts/J, stock no. 002287) mice were obtained from the Jackson Laboratory. All mice were between 5–8 weeks of age at the start of experimental work. Mice were infected via the lateral tail vein with 3x10^7^ amastigotes of the Ethiopian strain of *L*. *donovani* (LV9) or tandem Tomato fluorescent protein expressing *L*. *donovani* (tdTom-*L*. *donovani*). Spleen parasite burden was expressed as Leishman-Donovan units (LDU), where LDU was equal to the number of parasites/1000 host nuclei multiplied by the organ weight in milligrams. BM parasite burden was determined as the number of parasites/1000 host nuclei.

### Cell isolations and phenotypic characterization

Cell suspensions from spleen and BM (tibias and fibulas) were obtained as described previously [21]. For phenotypic analysis and FACS (Fluorescence-activated cell sorting) purification, BM cells and splenocytes were stained with a lineage marker cocktail (CD3e (145-2C11), Ly-6G and Ly-6C (RB6-8C5), TER-119 (TER-119), CD45R (RA3-6B2), and CD11b (M1/70)), Sca1 (D7), cKit (2B8), CD48 (HM48-1), CD34 (RAM34), FLT3 (A2F10), IL-7Rα (A7R34), CD45 (30-F11), CD150 (TC15-12F12.2), CD45.1 (A20), CD45.2 (104), CD3e (145-2C11 and UCHT1), CD4 (RM4-5 and 4SM95), CD8β (H35-17.2), CD11c (N418), CD11b (M1/70), MHC-II (M5/114.15.2), F4/80 (BM8), CD16/32 (93), TNFR1a (TR75-89), TNFR1b (55R-286), TNF (MP6-XT22), IFNγ (XMG1.2), GATA3 (16E10A23), Ki67 (MOPC-21), CD62L (MEL-14), CD44 (IM7), Ly6C (AL-21), TCRγδ (GL3) and CD49b (DX5). HSPCs were assigned based on criteria shown in **S1 Table**. Negative controls were stained with matched-isotype controls and dead cells were excluded using LIVE/DEAD Fixable Dead Cell Stains (Thermo Fisher Scientific). For intracellular staining with cytokines cell suspensions were stimulated with Phorbol-12-myristate-13-acetate (PMA) (Sigma-Aldrich) and ionomycin (Sigma-Aldrich) [63]. Analyses were performed either in the BD LSR Fortessa X-20 (BD Biosciences) or the CyAn ADP analyser (Beckman Coulter). MoFlo Astrios (Beckman Coulter) was used to perform sorting (to > 95% purity). Data was analysed with FlowJo software (TreeStar).

### Adoptive transfers and serial transfer of HSCs

BM cells from primary donors or from previously chimeric mice were sort purified as CD45^+^ Lin^-^ Sca1^+^ cKit^+^ CD150^+^ CD48^-^ CD34^-^ cells (LT-HSCs) or CD45^+^ Lin^-^ cells (enriched for HSPCs) and transplanted into lethally irradiated recipient mice (two doses of 550 rad, 24h apart). In non-competitive adoptive transfer experiments, radio-protective BM cells were transferred together with the sorted donor cells. For mixed BM chimeras, recipients received 1x10^6^ BM cells from each donor strain post-irradiation. Mice were infected at 7–9 weeks of chimerism. For the transplant of CD4^+^ T cells, 6x10^5^ of sort-purified splenic CD45^+^CD4^+^CD3^+^CD8^-^B220^-^TCRγδ^-^CD49b^-^ cells were transplanted into B6.*Rag2*^-/-^.CD45.1Cg recipient mice.

### Statistical analysis

Statistical analyses were performed by parametric or non-parametric tests, selected based on the distribution of the raw data. The comparisons between experimental groups were performed using student Unpaired t test, Mann-Whitney and one-way ANOVA. The analysis of population distribution was performed using Chi-square test. All analyses were conducted using GraphPad InStat (version 6) software (GraphPad).

## Supporting information

S1 Fig HSPCs from infected mice showed impaired engraftment in BM and decreased reconstitution of the periphery. Relates to Fig 2A–2D (A), representative dot plots of gating strategy used to segregate between recipient (CD45.1) and donor cells (CD45.2) →B6.CD45.1 x CD45.2_F1_ chimeras; control (left), transplanted (right). Relates to Fig 2E and 2F, (B) frequency of mature splenic haematopoietic cells within donor cells in recipient mice 16 weeks after transfer of 160 CD45.2 LT-HSCs (LSK CD150^+^ CD34^-^ CD48^-^) purified from naive (light squares) or day 28 infected (dark grey squares) mice. Relates to Fig 2G and 2H; analysis performed 24 weeks after transplant into B6 CD45.1 lethally irradiated mice of radiation protective total BM cells (3.5x10^5^) and 50 CD45.2 HSCs (LSK CD150^+^ CD34^-^ CD48^-^ cells) sort purified from CD45.1 recipient mice previously adoptively transferred with CD45.2 HSCs from mice naive or day 28 infected mice to lethally irradiated CD45.1 recipient mice for 16 weeks: (C) number of CMPs, GMPs, MEPs and CLPs within each donor compartment in the BM of non-infected recipient mice, (D) number of mature hematopoietic cells: B cells, T cells and CD11b^+^ cells (myeloid cells) within donor cells in the spleen of recipient mice. Absolute numbers were calculated from two femurs and two tibias for each mouse. Data shown as scatter plot and mean bar. Comparisons were made between naive donor cells (n = 4) and infected donor cells (n = 3–4). *p* values were determined using unpaired t test: *p ≤ 0.05, **p ≤0.01, ****p ≤0.001. (E) Representative dot plots gated in BM lineage^neg^ cells (left) and LT-HSCs (right) to assess parasite infection in mice infected for 28 days with LV9.TdTom (n = 5).
(TIF)

S2 Fig Enhanced proliferation of HSCs was associated with increased levels of GATA-3 following *L. donovani* infection. (A) Representative dot plots of gating to select GATA-3^+^ cells in LSK CD150^+^ cells (enriched for non-committed progenitors). (B) Frequency of cells expressing Ki67 and GATA-3 within LSK CD150^+^ CD48^-^ cells (enriched for LT-HSCs). Data from two independent experiments (n = 8 per group) presented as scatter plot and mean bar; *p* values were determined using unpaired t test: *p ≤ 0.05, **p ≤0.01, ****p ≤0.001. (C) Frequency distribution of LSK CD150^+^ CD48^-^ sub populations based on Ki67 and GATA-3 expression. Mean from two independent experiments (n = 8 per group): *p ≤ 0.05, **p ≤0.01, ***p ≤0.001, ***p ≤0.0001; Chi-square test.
(TIF)

S3 Fig Lack of intrinsic IFNγ receptor signalling affects development of CD11b^+^F4/80^hi^ cells following infection. Relates to Fig 7 (A) Frequency of BM lineage-committed progenitors in naïve (light symbols) and infected (dark grey symbols) mice derived from HSCs of B6.WT or B6.IFNγR2^−/−^ origin (squares and triangles, respectively). (B-E) Frequencies of: BM B cells (B), BM myeloid subsets (C), splenic B cells (D), and splenic myeloid cells (E) within each donor population. Analyses were performed 12 weeks after transplant of BM cells from CD45.2 *Ifnγr2*^−/−^ mice and CD45.1 WT mice (50:50) to lethally irradiated CD45.1 recipients. Data was presented as scatter plot and mean bar (n = 4 per group); *p ≤ 0.05, **p ≤0.01, ***p ≤0.001 and ****p ≤0.0001; One-way Anova followed by Tukey’s multiple comparisons test.
(TIF)

S4 Fig TNFR expression increases on BM cells following *L. donovani* infection. Relates to Fig 8 (A) Frequency of BM CD45^+^ Lineage^+^ cells and Lineage- cKit^+^ cells expressing TNFR1a. (b) Frequency of BM HSPCs populations expressing TNF-R1a. (c) MFI of TNF-R1a on HSPCs. (D) Representative histogram of TNF-R1a expression on LSK CD150^+^ cells. (E) Frequency of BM CD45^+^ Lineage^+^ cells and Lineage- cKit^+^ cells expressing TNF-R1b. (F) Frequency of BM HSPCs populations expressing TNF-R1b (G) MFI of TNF-R1b expression on HSPCs. (H) Representative histogram of TNF-R1b expression on LSK CD150^+^ cells. Data from one experiment as Mean ± SD (n = 5 per group); *p ≤ 0.05, **p ≤0.01, ***p ≤0.001 and ****p ≤0.0001; unpaired t test.
(TIF)

S5 Fig TNF receptor signalling is not required for B cell and myeloid cell development. Relates to Fig 8. (A) Frequency of lineage-committed progenitors, (B) B cells and CD11b^+^ cells in the BM of naïve and infected recipient mice derived from HSCs of B6.WT (squares) or B6 *Tnfrsf1*-dKO (triangle) origin within donor cells. (C) Frequency of myeloid cells, (D) B cells (E) and T cells in the spleen of naïve and infected recipient mice derived from HSCs of B6.WT or B6 *Tnfrsf1*-dKO origin within donor cells. (F) Number of T cells in the spleen of recipient mice derived from HSCs of B6.WT or B6 *Tnfrsf1*-dKO origin within donor cells. Analyses were performed 13 weeks after transplant of BM cells from CD45.2 TNFRdKO mice and CD45.1 WT mice (50:50) to lethally irradiated CD45.1 recipients. Data from two independent experiments was presented as Scatter-plot and mean (n = 8–9 per group); *p ≤ 0.05, **p ≤0.01, ***p ≤0.001 and ****p ≤0.0001; One-way Anova followed by Tukey’s multiple comparisons test.
(TIF)

S6 Fig Proposed model for development of pathogenic BM CD4^+^ T cells. Following *L*. *donovani* infection, proliferating LT-HSCs and onward multipotent progenitors expand greatly at the expense of LT-HSCs in G0, leading to functional exhaustion, as demonstrated by serial transfer. CD4^+^ T cells mediate LT-HSC exhaustion through an INFγ-dependent mechanism. However, the expansion of pathogenic CD4^+^ T cells secreting INFγ^+^ is limited in the absence of T cell-intrinsic TNF receptor signaling, indicating that TNF indirectly modulates LT-HSCs exhaustion during chronic infection in *L*. *donovani*.
(TIF)

S1 Table Surface markers used to characterize HSPCs by flow cytometry analysis. (TIF)
