# Supplementary figures and images for: TNF signalling drives expansion of bone marrow CD4+ T cells responsible for HSC exhaustion in experimental visceral leishmaniasis
Source: PLoS Pathog. 2017 Jul 3;13(7):e1006465. doi: 10.1371/journal.ppat.1006465 (PMC5510901; doi:10.1371/journal.ppat.1006465)

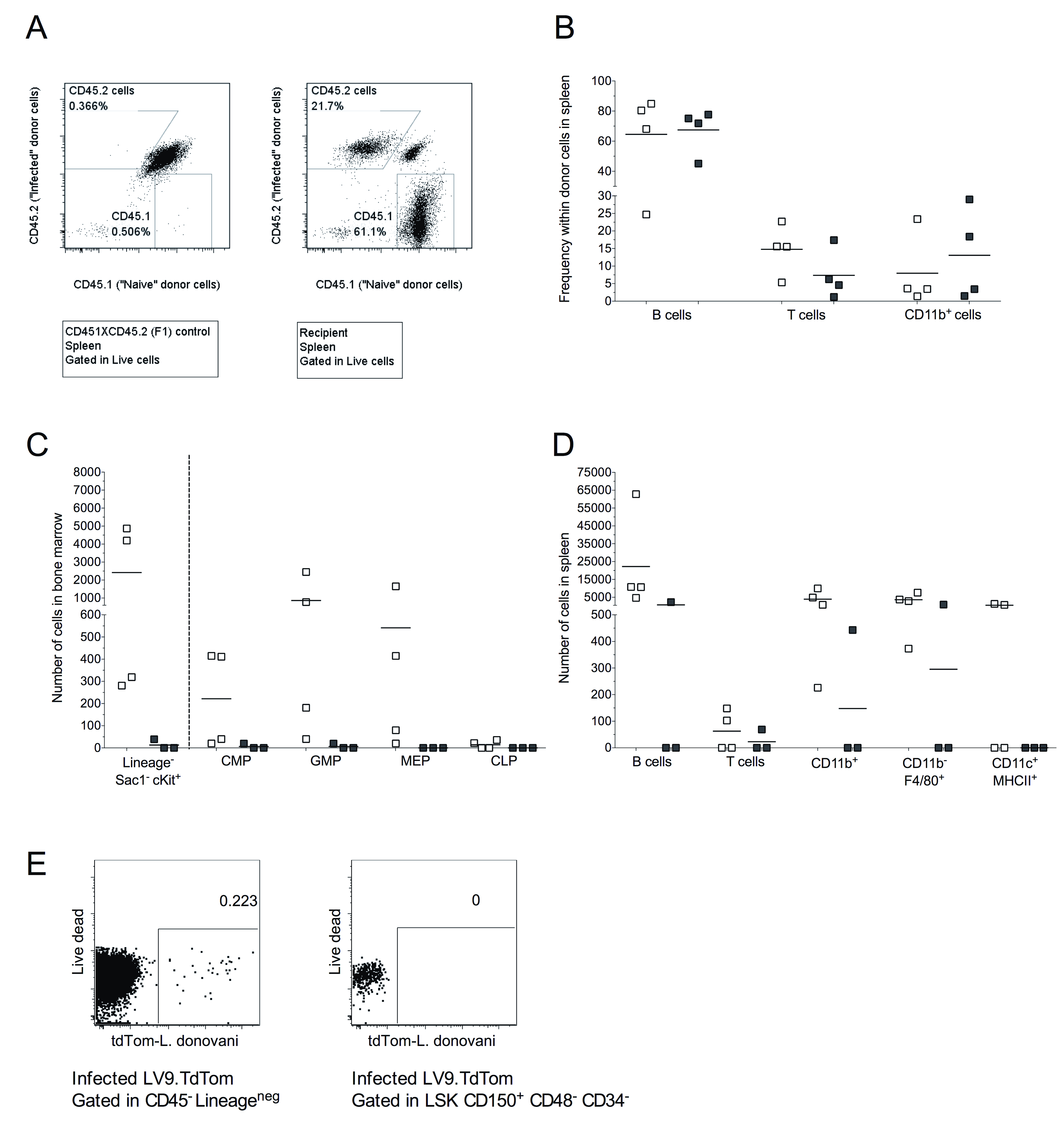

Supplement: S1 Fig — Relates to Fig 2A–2D (A), representative dot plots of gating strategy used to segregate between recipient (CD45.1) and donor cells (CD45.2) →B6.CD45.1 x CD45.2F1 chimeras; control (left), transplanted (right). Relates to Fig 2E and 2F, (B) frequency of mature splenic haematopoietic cells within donor cells in recipient mice 16 weeks after transfer of 160 CD45.2 LT-HSCs (LSK CD150+ CD34- CD48-) purified from naive (light squares) or day 28 infected (dark grey squares) mice. Relates to Fig 2G and 2H; analysis performed 24 weeks after transplant into B6 CD45.1 lethally irradiated mice of radiation protective total BM cells (3.5x10^5^) and 50 CD45.2 HSCs (LSK CD150+ CD34- CD48- cells) sort purified from CD45.1 recipient mice previously adoptively transferred with CD45.2 HSCs from mice naive or day 28 infected mice to lethally irradiated CD45.1 recipient mice for 16 weeks: (C) number of CMPs, GMPs, MEPs and CLPs within each donor compartment in the BM of non-infected recipient mice, (D) number of mature hematopoietic cells: B cells, T cells and CD11b+ cells (myeloid cells) within donor cells in the spleen of recipient mice. Absolute numbers were calculated from two femurs and two tibias for each mouse. Data shown as scatter plot and mean bar. Comparisons were made between naive donor cells (n = 4) and infected donor cells (n = 3–4). p values were determined using unpaired t test: *p ≤ 0.05, **p ≤0.01, ****p ≤0.001. (E) Representative dot plots gated in BM lineageneg cells (left) and LT-HSCs (right) to assess parasite infection in mice infected for 28 days with LV9.TdTom (n = 5). (TIF) [file ppat.1006465.s001.tif]

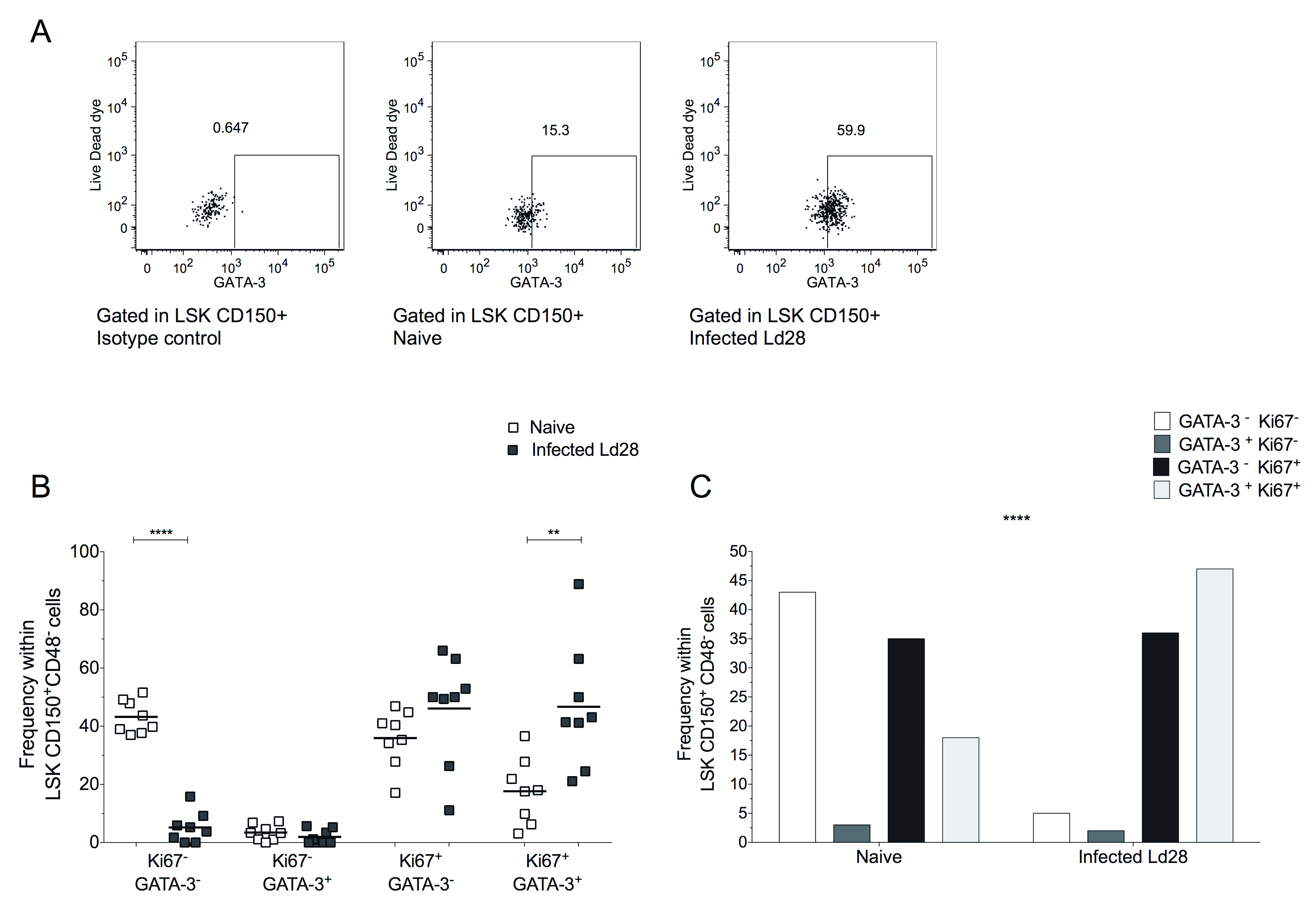

Supplement: S2 Fig — (A) Representative dot plots of gating to select GATA-3+ cells in LSK CD150+ cells (enriched for non-committed progenitors). (B) Frequency of cells expressing Ki67 and GATA-3 within LSK CD150+ CD48- cells (enriched for LT-HSCs). Data from two independent experiments (n = 8 per group) presented as scatter plot and mean bar; p values were determined using unpaired t test: *p ≤ 0.05, **p ≤0.01, ****p ≤0.001. (C) Frequency distribution of LSK CD150+ CD48- sub populations based on Ki67 and GATA-3 expression. Mean from two independent experiments (n = 8 per group): *p ≤ 0.05, **p ≤0.01, ***p ≤0.001, ***p ≤0.0001; Chi-square test. (TIF) [file ppat.1006465.s002.tif]

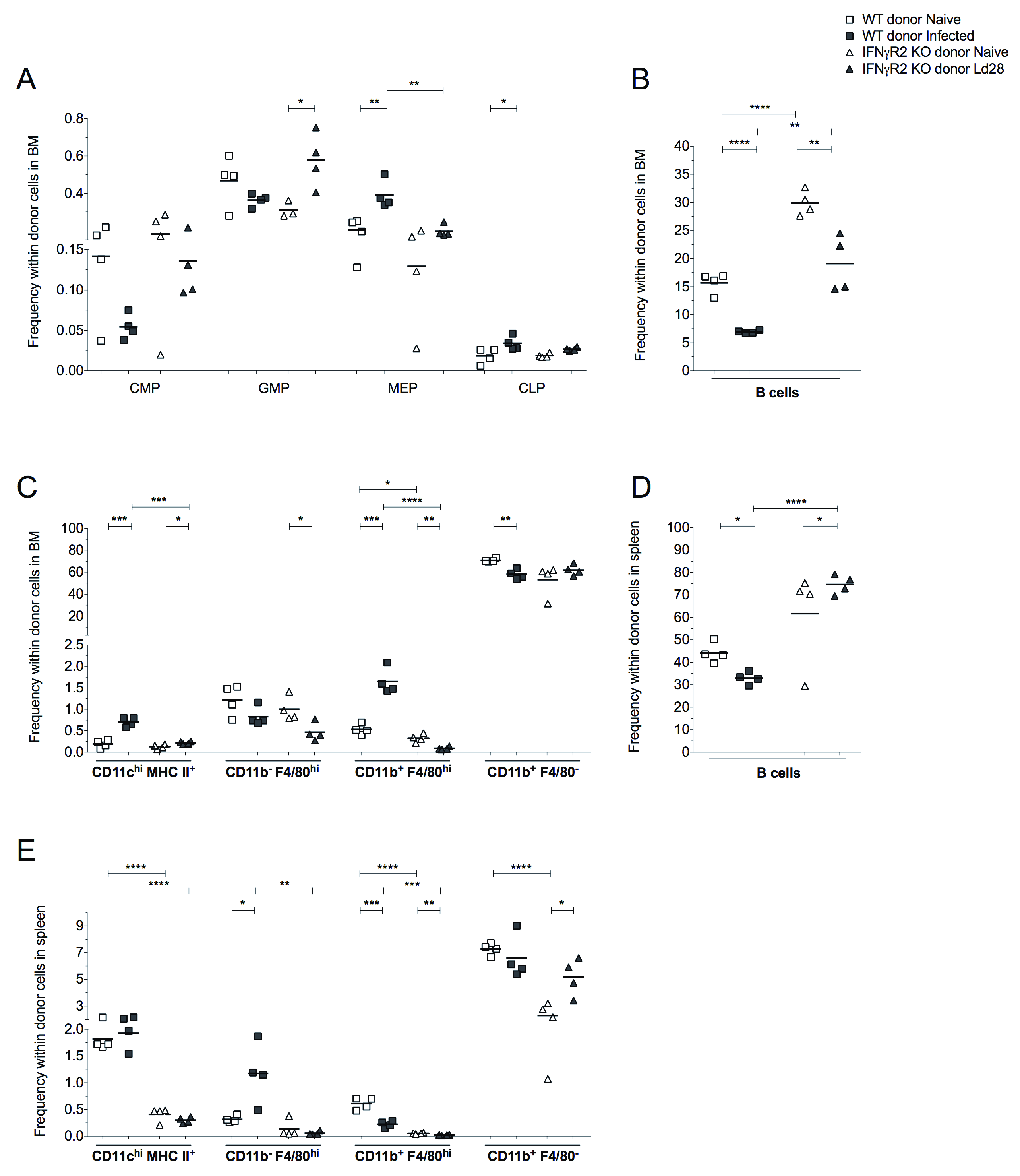

Supplement: S3 Fig — Relates to Fig 7 (A) Frequency of BM lineage-committed progenitors in naïve (light symbols) and infected (dark grey symbols) mice derived from HSCs of B6.WT or B6.IFNγR2−/− origin (squares and triangles, respectively). (B-E) Frequencies of: BM B cells (B), BM myeloid subsets (C), splenic B cells (D), and splenic myeloid cells (E) within each donor population. Analyses were performed 12 weeks after transplant of BM cells from CD45.2 Ifnγr2−/− mice and CD45.1 WT mice (50:50) to lethally irradiated CD45.1 recipients. Data was presented as scatter plot and mean bar (n = 4 per group); *p ≤ 0.05, **p ≤0.01, ***p ≤0.001 and ****p ≤0.0001; One-way Anova followed by Tukey’s multiple comparisons test. (TIF) [file ppat.1006465.s003.tif]

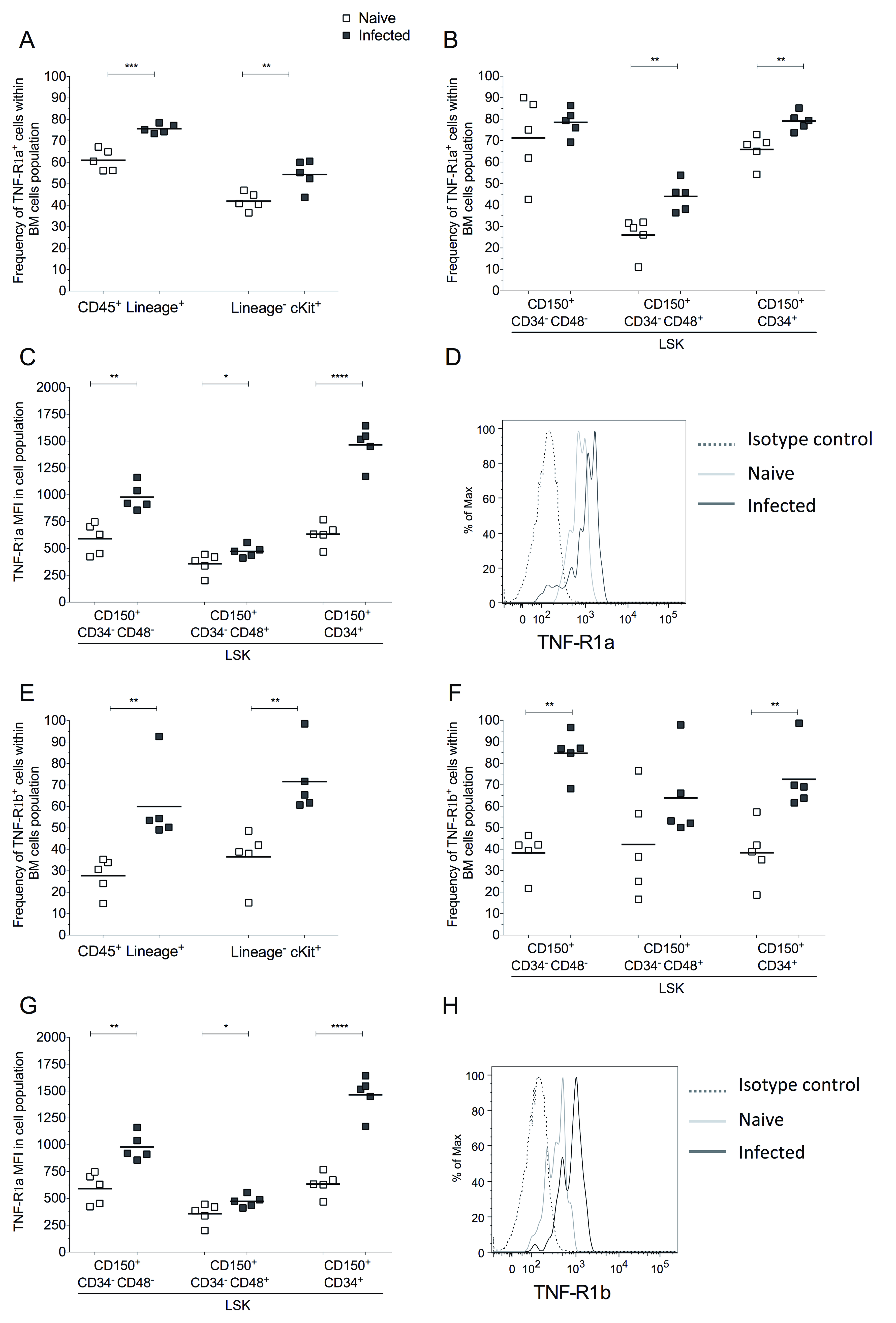

Supplement: S4 Fig — Relates to Fig 8 (A) Frequency of BM CD45+ Lineage+ cells and Lineage- cKit+ cells expressing TNFR1a. (b) Frequency of BM HSPCs populations expressing TNF-R1a. (c) MFI of TNF-R1a on HSPCs. (D) Representative histogram of TNF-R1a expression on LSK CD150+ cells. (E) Frequency of BM CD45+ Lineage+ cells and Lineage- cKit+ cells expressing TNF-R1b. (F) Frequency of BM HSPCs populations expressing TNF-R1b (G) MFI of TNF-R1b expression on HSPCs. (H) Representative histogram of TNF-R1b expression on LSK CD150+ cells. Data from one experiment as Mean ± SD (n = 5 per group); *p ≤ 0.05, **p ≤0.01, ***p ≤0.001 and ****p ≤0.0001; unpaired t test. (TIF) [file ppat.1006465.s004.tif]

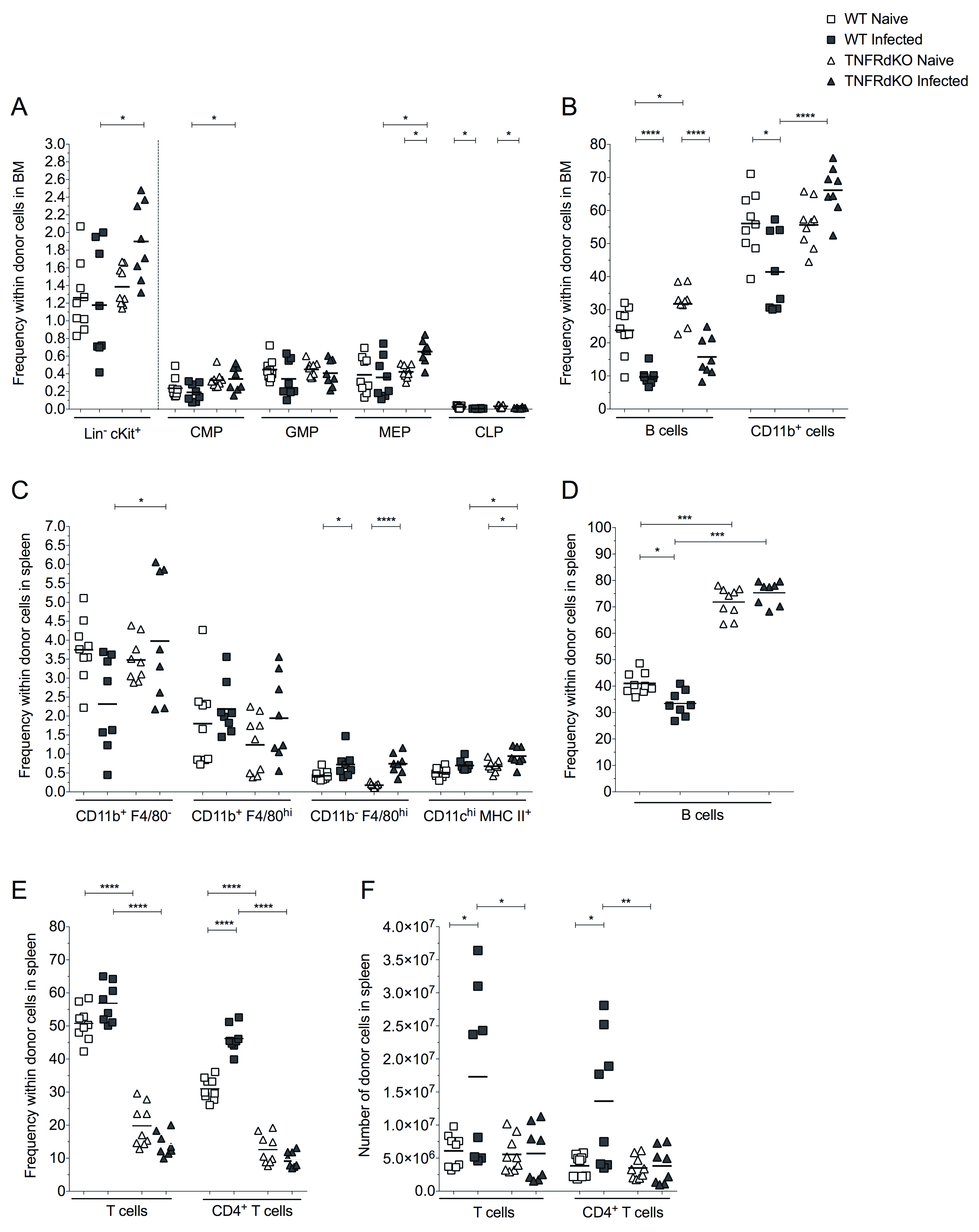

Supplement: S5 Fig — Relates to Fig 8. (A) Frequency of lineage-committed progenitors, (B) B cells and CD11b+ cells in the BM of naïve and infected recipient mice derived from HSCs of B6.WT (squares) or B6 Tnfrsf1-dKO (triangle) origin within donor cells. (C) Frequency of myeloid cells, (D) B cells (E) and T cells in the spleen of naïve and infected recipient mice derived from HSCs of B6.WT or B6 Tnfrsf1-dKO origin within donor cells. (F) Number of T cells in the spleen of recipient mice derived from HSCs of B6.WT or B6 Tnfrsf1-dKO origin within donor cells. Analyses were performed 13 weeks after transplant of BM cells from CD45.2 TNFRdKO mice and CD45.1 WT mice (50:50) to lethally irradiated CD45.1 recipients. Data from two independent experiments was presented as Scatter-plot and mean (n = 8–9 per group); *p ≤ 0.05, **p ≤0.01, ***p ≤0.001 and ****p ≤0.0001; One-way Anova followed by Tukey’s multiple comparisons test. (TIF) [file ppat.1006465.s005.tif]

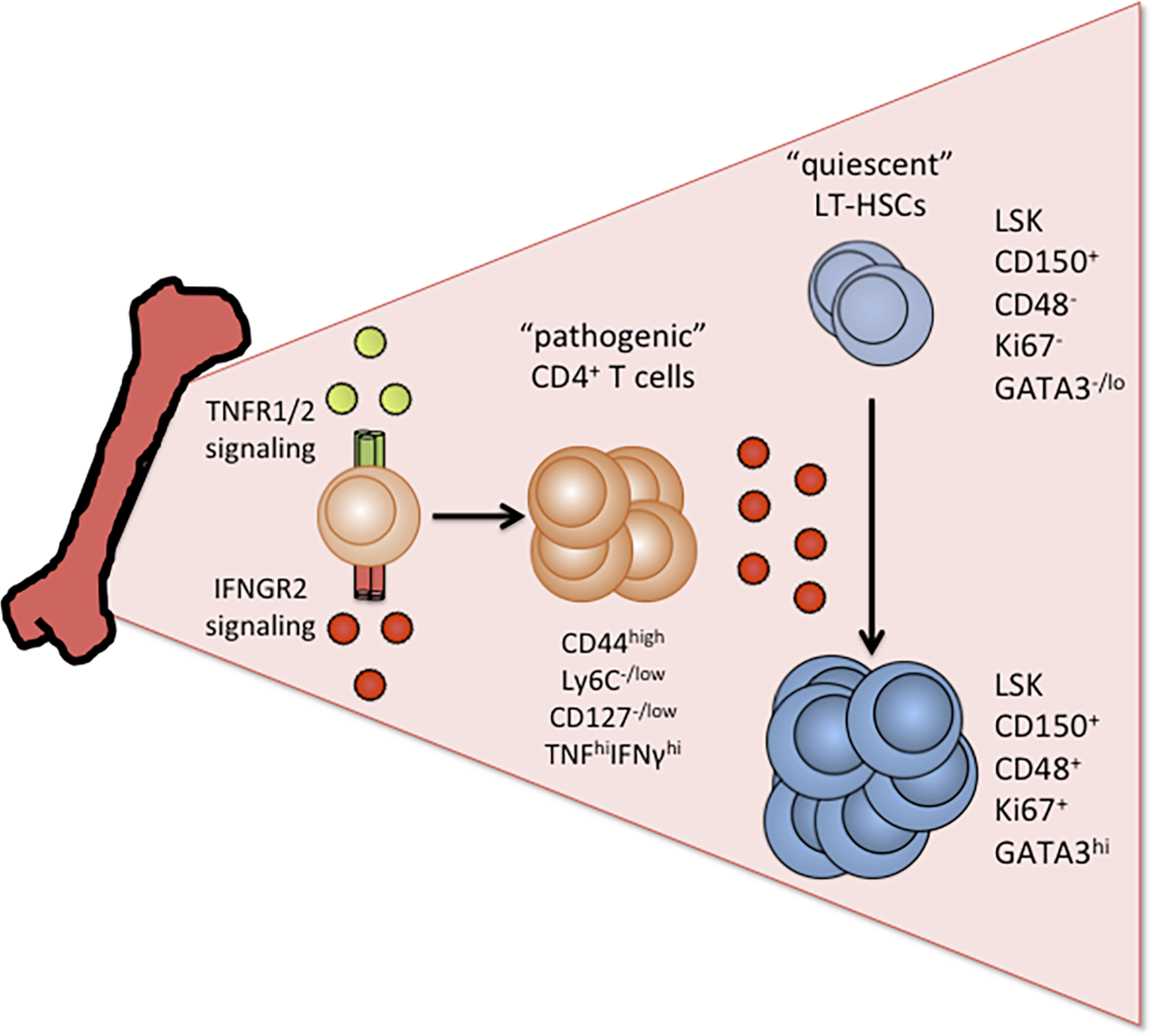

Supplement: S6 Fig — Following L. donovani infection, proliferating LT-HSCs and onward multipotent progenitors expand greatly at the expense of LT-HSCs in G0, leading to functional exhaustion, as demonstrated by serial transfer. CD4+ T cells mediate LT-HSC exhaustion through an INFγ-dependent mechanism. However, the expansion of pathogenic CD4+ T cells secreting INFγ+ is limited in the absence of T cell-intrinsic TNF receptor signaling, indicating that TNF indirectly modulates LT-HSCs exhaustion during chronic infection in L. donovani. (TIF) [file ppat.1006465.s006.tif]

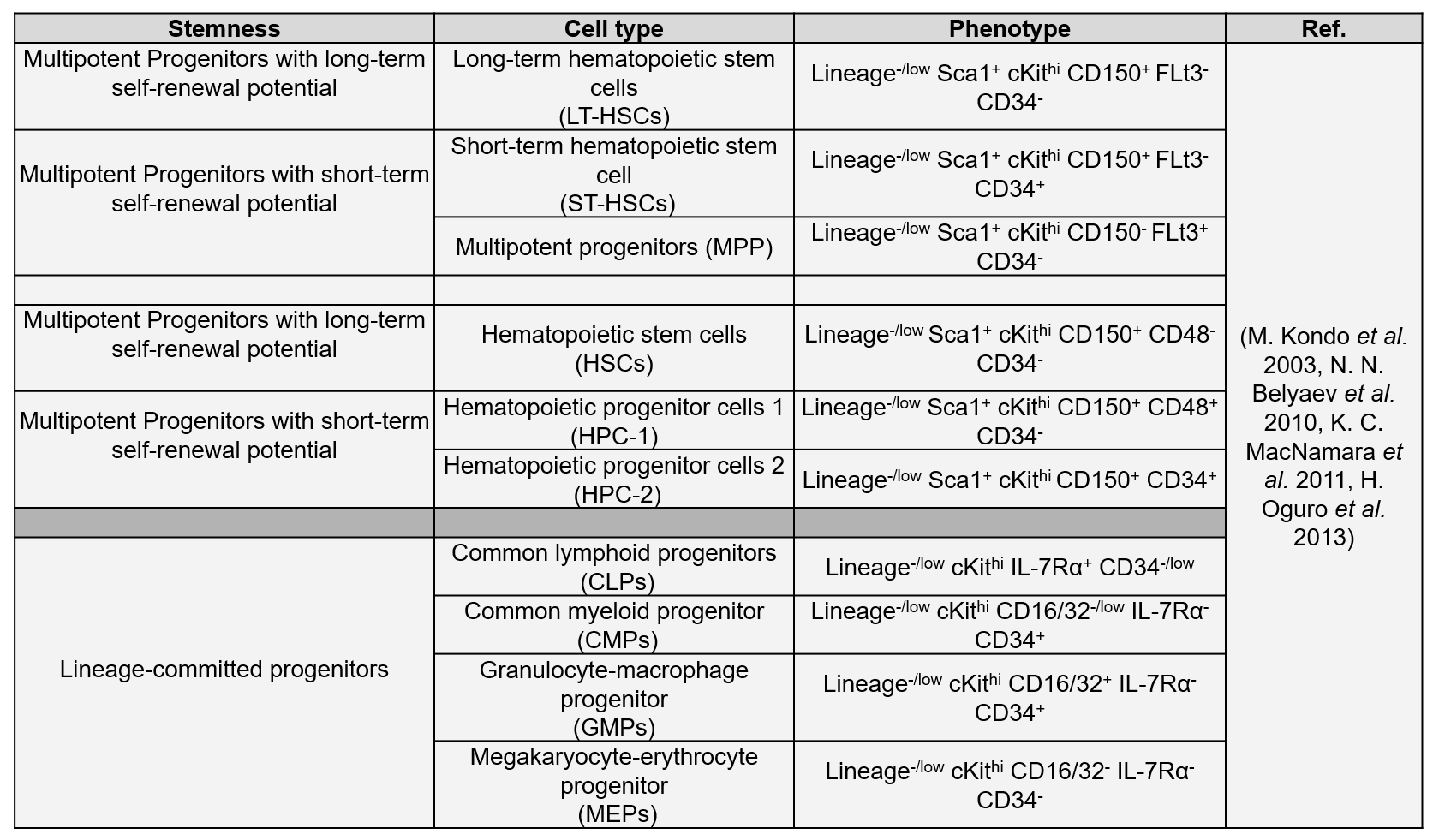

Supplement: S1 Table — (TIF) [file ppat.1006465.s007.tif]
